# Supplementary material for: Combination of Biochar and Trichoderma harzianum Can Improve the Phytoremediation Efficiency of Brassica juncea and the Rhizosphere Micro-Ecology in Cadmium and Arsenic Contaminated Soil
Source: Plants (Basel). 2023 Aug 14;12(16):2939. doi: 10.3390/plants12162939 (PMC10458205; doi:10.3390/plants12162939)
Supplement: Supplementary file 1 [file plants-12-02939-s001.zip › plants-2530449-supplementary.pdf]

Supplementary Information

Effects of combined biochar and *Trichoderma harzianum* on improving the cadmium and arsenic phytoremediation efficiency of *Brassica juncea* and the response of the rhizosphere soil micro-ecology

Shaoxiong Yao[a], Beibei Zhou[a]*, Manli Duan[a], Tao Cao[a], Zhaoquan Wen[a], Xiaopeng Chen[a], Hui Wang[a], Min Wang[a], Wen Cheng[a], Hongyan Zhu[a], Qiang Yang[b], Yujin Li[b]

[a] State Key Laboratory of Eco-Hydraulics in Northwest Arid Region of China, Xi'an University of Technology, Xi'an 710048, China; 580231.ysx@gmail.com (S.Y.); manli0815@163.com (M.D.); 15102711614@163.com (T.C.); blank4634@163.com (Z.W.); man_xp@sina.cn (X.C.); wanghui306@xaut.edu.cn (H.W.); wangmin@xaut.edu.cn (M.W.); wencheng@xaut.edu.cn (W.C.); zhy@xaut.edu.cn (H.Z.)

[b] PowerChina Northwest Engineering Corporation Limited, Xi'an 710065, China; yangqiang@nwh.cn (Q.Y.); liyujin@nwh.cn (Y.L.).

* Corresponding author. E-mail address: happyangle222@aliyun.com (B. B. Zhou)

Table S1 Soil physicochemical characteristics in the experimental field

Table S2 Plant shoots and roots biomass (mean ± S.E., n=3)

Table S3 Variation in soil physicochemical properties (pH, EC, CEC, AN, AP, AK, OM) in the inter-root, 15cm, 30cm and 45cm soil layers under different restoration treatments (mean ± S.E., n=3)

Table S4 Rhizosphere soil microbial community α-diversity index (mean ± S.E., n=3)

Figure S1 Rarefaction curves of bacteria(A) and fungi(B) at the 97% sequence similarity level.

Figure S2 Heatmap of the 30 most abundant genera for bacteria (A) and fungi (B) across all 6 samples in rhizosphere soil.

Figure S3 Pearson correlation between rhizosphere soil physicochemical properties, enzyme activity and plant potentially toxic elements accumulation.

Table S1. Soil physicochemical characteristics in the experimental field

**Soil parameter**	**Value**		
	**0-15 cm deep soil**	**15-30 cm deep soil**	**30-45 cm deep soil**
pH	8.33	8.28	8.39
Organic matter (OM, g kg^{-1})	16.03	15.32	17.14
Cation exchange capacity (CEC, cmol kg^{-1})	22.55	20.65	21.43
Available nitrogen (AN, mg kg^{-1})	40.34	45.21	38.75
Available potassium (AK, mg kg^{-1})	140.30	130.25	105.95
Available phosphorus (AP, mg kg^{-1})	3.56	3.12	3.29
Total Cd (mg kg^{-1})	1.54	1.64	1.30
Total As (mg kg^{-1})	24.42	44.36	46.93
Total Hg (mg kg^{-1})	5.89	3.57	2.93
Total Pb (mg kg^{-1})	38.58	38.61	35.45
Total Cu (mg kg^{-1})	43.31	42.80	45.28
Total Cr (mg kg^{-1})	209.59	194.51	233.37

Table S2. Biochar physicochemical characteristics in this experimental

	pH	Diameter (mm)	Water content (%)	Ash content (%)	Total nitrogen (g kg^{-1})	Total phosphorus (g kg^{-1})	Total potassium (g kg^{-1})
Biochar	7.23	1	1.02	4.4	7.32	3.24	17.06

Table S3. Plant shoots and roots biomass (mean ± S.E., n=3)

Treatment	Shoots biomass	Roots biomass	Chlorophyll a	Chlorophyll b	Total chlorophyll
CK	0.588 ±0.024f	0.064 ±0.005f	0.633±0.05d	0.482±0.04d	1.111±0.06b
B	1.784 ±0.069b	0.110 ±0.004d	0.757±0.04c	0.356±0.02e	1.109±0.04b
T1	0.818 ±0.031e	0.153 ±0.006b	1.239±0.09a	1.081±0.05a	1.155±0.06b
T2	1.038 ±0.060d	0.075 ±0.005e	0.755±0.03c	0.395±0.02e	1.146±0.04b
T1B	1.328 ±0.049c	0.130 ±0.005c	0.645±0.04d	0.925±0.05b	1.563±0.08a
T2B	2.012 ±0.138a	0.183 ±0.012a	0.869±0.05b	0.817±0.05c	1.680±0.12a

CK, *Brassica juncea* alone; B, *Brassica juncea* + biochar; T1, *Brassica juncea* + 4.5 g m^{-2} TH; T2, *Brassica juncea* + 9 g m^{-2} TH; T1B, *Brassica juncea* + biochar + 4.5 g m^{-2} TH; T2B, *Brassica juncea* + biochar + 9 g m^{-2} TH. Different letters in each column represent significant differences at $p < 0.05$.

Table S4. Variation in soil physicochemical properties (pH, EC, CEC, AN, AP, AK, OM) in the inter-root, 15cm, 30cm and 45cm soil layers under different restoration treatments (mean ± S.E., n=3)

Hierarchical processing	pH	EC (mS cm^{-1})	CEC (cmol kg^{-1})	AN (mg kg^{-1})	AP (mg kg^{-1})	AK (mg kg^{-1})	OM (g kg^{-1})
Rhizosphere soil							
CK	8.32±0.38a	84.64±5.31b	27.30±1.38d	40.42±2.97c	7.38±0.27d	46.44±2.15d	21.61±0.85c
B	8.35±0.39a	89.08±5.43b	34.43±1.73c	44.62±2.46b	14.74±0.56b	106.59±5.31a	23.32±0.85b
T1	8.32±0.41a	78.88±5.12c	29.51±1.57d	33.34±1.98d	8.24±0.31d	60.63±2.78c	13.91±0.55
T2	8.20±0.39a	76.86±4.89c	37.53±1.91b	42.18±2.52bc	11.86±0.47c	66.46±3.18c	20.49±0.76c
T1B	8.26±0.40a	136.65±8.88a	38.94±2.08b	55.35±3.14a	15.14±1.00b	79.17±3.88b	23.94±0.85b
T2B	8.04±0.40a	85.65±5.53b	48.70±2.55a	51.07±2.98a	23.05±0.49a	103.26±4.77a	30.17±0.76a
0-15cm deep soil							
CK	8.67±0.39a	78.65±5.10bc	26.82±1.42d	39.51±3.56d	5.39±0.21d	44.02±2.08d	18.04±0.69d
B	8.70±0.42a	82.22±5.16b	34.33±1.72b	43.21±2.57c	4.98±0.18e	53.02±2.63b	22.08±0.84b
T1	8.67±0.41a	81.00±5.10b	30.43±1.65c	42.50±2.46c	5.46±0.21d	46.56±2.23d	17.09±0.68d
T2	8.54±0.42a	74.06±4.58c	30.72±1.62c	49.59±2.78b	6.14±0.23c	48.53±2.33c	20.13±0.77c
T1B	8.60±0.41a	96.40±5.93a	36.42±1.92b	57.38±3.21a	6.83±0.25b	62.87±2.98a	19.96±0.71c
T2B	8.38±0.38a	78.75±5.01bc	42.85±2.15a	46.75±2.77b	7.49±0.19a	63.33±3.02a	27.80±0.69a
15-30cm deep soil							
CK	8.69±0.42a	77.10±4.97c	28.73±1.53c	65.21±3.86a	0.82±0.03e	34.78±1.63d	14.88±0.56c
B	8.64±0.43a	80.60±4.94b	35.71±1.90b	43.92±2.52c	2.58±0.10c	21.55±1.08e	21.77±0.78a
T1	8.74±0.41a	79.40±4.84bc	24.51±1.27d	27.77±1.66d	1.52±0.06d	49.26±2.33b	18.10±0.71b
T2	8.59±0.40a	72.60±4.68d	22.39±1.13d	39.18±2.34cd	2.69±0.09c	40.46±1.99c	17.04±0.60b
T1B	8.77±0.40a	94.50±6.08a	38.77±2.09a	49.57±2.82b	3.23±0.13b	65.26±3.13a	13.41±0.52d
T2B	8.73±0.42a	77.20±4.87c	33.34±1.73bc	42.86±2.51c	4.96±0.04a	47.61±2.23b	13.95±0.51d
30-45cm deep soil							
CK	8.71±0.40a	81.10±5.08bc	26.74±1.41c	47.07±2.72a	1.60±0.06f	34.74±1.73c	10.19±0.39c
B	8.73±0.41a	78.20±5.05cd	30.47±1.64b	28.88±1.06d	6.44±0.25c	34.53±1.62c	16.93±0.67a
T1	8.64±0.41a	87.60±5.41a	21.04±1.13de	25.31±1.50e	3.04±0.12e	39.78±1.81bc	14.72±0.58b
T2	8.63±0.41a	73.10±4.56d	19.36±0.99e	34.39±2.01bc	9.31±0.35a	21.47±1.03e	9.13±0.35d
T1B	8.89±0.43a	84.00±5.37bc	40.84±2.17a	32.57±1.79c	7.12±0.28b	44.21±2.01b	15.93±0.62ab
T2B	8.72±0.42a	85.60±5.17b	22.63±1.24d	37.19±2.23b	3.52±0.13d	67.81±3.07a	13.95±0.55b

CK, *Brassica juncea* alone; B, *Brassica juncea* + biochar; T1, *Brassica juncea* + 4.5 g m^{-2} TH; T2, *Brassica juncea* + 9 g m^{-2} TH; T1B, *Brassica juncea* + biochar + 4.5 g m^{-2} TH; T2B, *Brassica juncea* + biochar + 9 g m^{-2} TH. EC, Electric Conductivity; CEC, Cation Exchange Capacity; OM, Organic Matter; AN, Available Nitrogen; AP, Available Phosphorus; AK, Available Potassium. Different letters in each column represent significant differences at $p < 0.05$.

Table S5. Rhizosphere soil microbial community α-diversity index (mean ± S.E., n=3)

Treatment	Observed species	Shannon	Simpson	Chao1	ACE	Goods coverage	PD-whole-tree
Bacteria							
CK	2490± 92.709c	9.070± 0.336a	0.993± 0.049a	3313.583± 194.327b	3365.046± 161.326bc	0.965± 0.062a	323.551± 16.963c
B	2366± 148.235c	9.183± 0.553a	0.996± 0.065a	2930.816± 128.822c	3023.597± 137.712d	0.971± 0.034a	306.412± 16.535cd
T1	2434± 123.818c	9.183± 0.343a	0.994± 0.034a	3124.490± 174.937bc	3279.747± 234.435cd	0.967± 0.060a	342.666± 23.421c
T2	1892± 105.922d	6.621± 0.253b	0.912± 0.055a	2365.789± 164.826d	2550.353± 138.612e	0.974± 0.035a	262.356± 15.770d
T1B	2899± 141.802b	9.443± 0.416a	0.993± 0.056a	3355.163± 167.028b	3705.366± 197.736b	0.965± 0.040a	442.681± 24.523b
T2B	3207± 107.960a	9.250± 0.514a	0.989± 0.050a	4302.027± 265.718a	4196.474± 165.109a	0.955± 0.054a	591.809± 38.150a
Fungi							
CK	977± 53.936c	5.951± 5.930c	0.96± 0.040a	1407.519± 82.801d	1446.18± 95.511d	0.991± 0.053a	390.32± 24.605d
B	1522± 76.308b	7.972± 7.920a	0.985± 0.042a	1636.026± 72.708c	1663.018± 88.360c	0.994± 0.071a	318.426± 16.752e
T1	1723± 109.675a	6.977± 6.920b	0.972± 0.043a	2416.004± 128.179a	2454.153± 92.410a	0.985± 0.043a	694.784± 27.735a
T2	1057± 73.675c	4.887± 4.750d	0.812± 0.027b	1493.269± 94.834cd	1541.528± 109.101cd	0.990± 0.031a	592.475± 34.276b
T1B	1781± 63.120a	7.467± 7.400ab	0.982± 0.037a	2226.385± 101.562b	2241.586± 118.568b	0.988± 0.049a	514.816± 19.266c
T2B	785± 46.695d	5.210± 5.160d	0.911± 0.062a	1050.044± 69.136e	1093.037± 51.324e	0.994± 0.062a	364.141± 22.792de

CK, *Brassica juncea* alone; B, *Brassica juncea* + biochar; T1, *Brassica juncea* + 4.5 g m^{-2} TH; T2, *Brassica juncea* + 9 g m^{-2} TH; T1B, *Brassica juncea* + biochar + 4.5 g m^{-2} TH; T2B, *Brassica juncea* + biochar + 9 g m^{-2} TH. Different letters in each column represent significant differences at $p<0.05$.

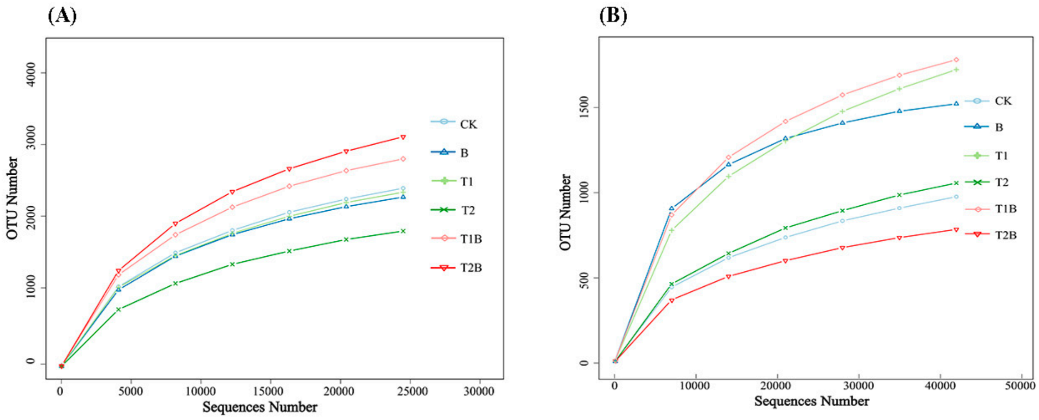

Figure S1. Rarefaction curves of bacteria(A) and fungi(B) at the 97% sequence similarity level. CK, *Brassica juncea* alone; B, *Brassica juncea* + biochar; T1, *Brassica juncea* + 4.5 g m^{-2} TH; T2, *Brassica juncea* + 9 g m^{-2} TH; T1B, *Brassica juncea* + biochar + 4.5 g m^{-2} TH; T2B, *Brassica juncea* + biochar + 9 g m^{-2} TH.

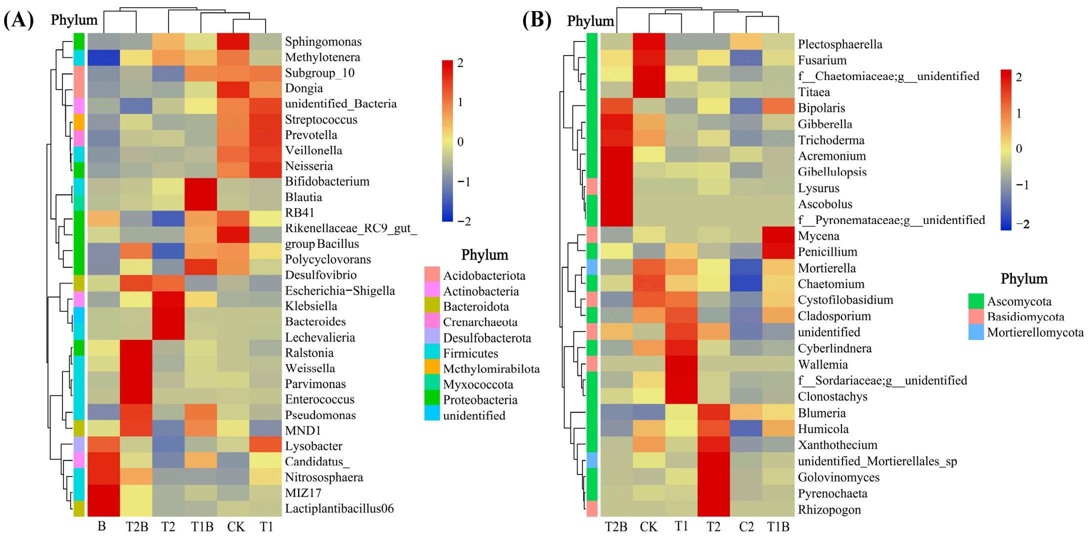

Figure S2. Heatmap of the 30 most abundant genera for bacteria (A) and fungi (B) across all 6 samples in rhizosphere soil. CK, *Brassica juncea* alone; B, *Brassica juncea* + biochar; T1, *Brassica juncea* + 4.5 g m^{-2} TH; T2, *Brassica juncea* + 9 g m^{-2} TH; T1B, *Brassica juncea* + biochar + 4.5 g m^{-2} TH; T2B, *Brassica juncea* + biochar + 9 g m^{-2} TH. Abundance is expressed as color intensity, reflecting the proportion of the total number of valid sequences in each group. Vertical: treatment; Horizontal: annotation information for species.

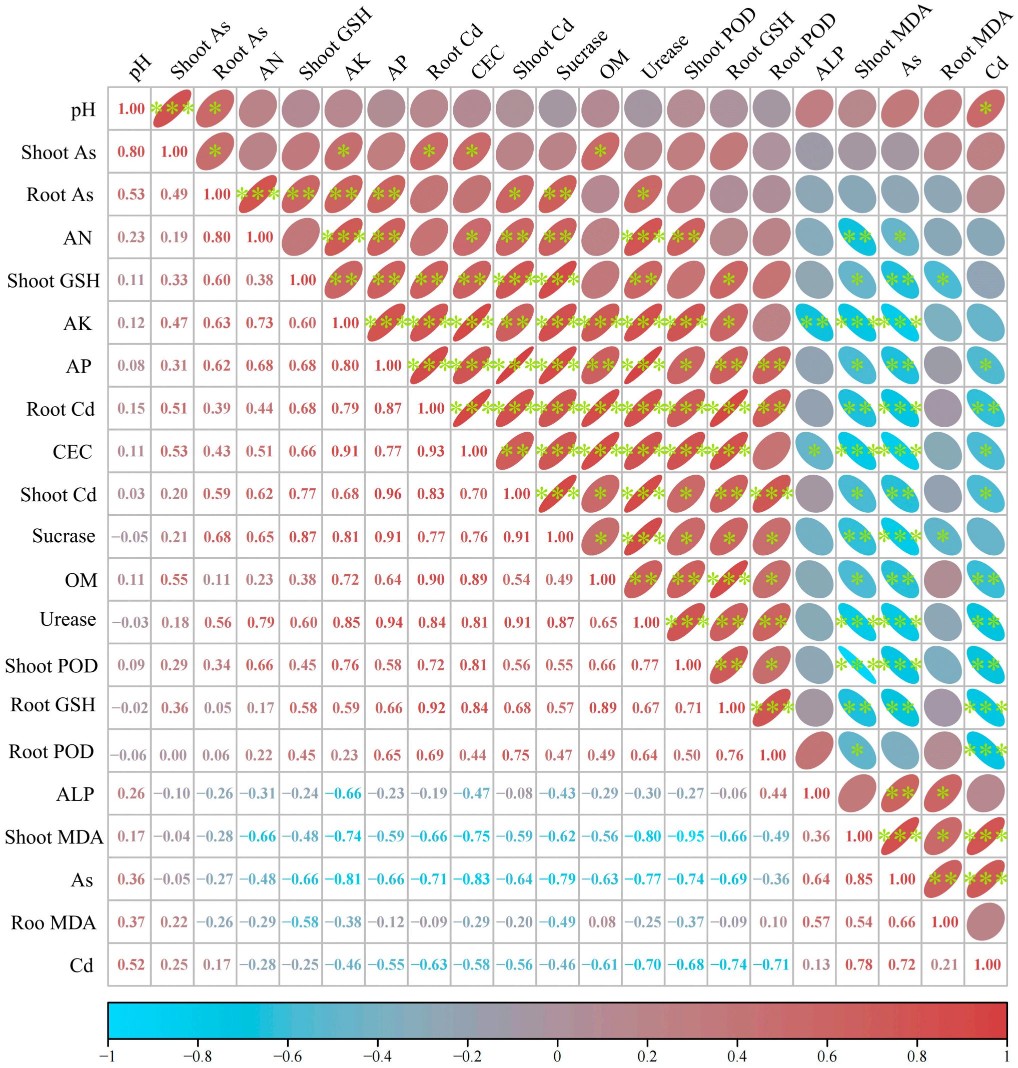

Figure S3. Pearson correlation between rhizosphere soil physicochemical properties, enzyme activity and plant heavy metal accumulation. The number represents the correlation coefficient between the parameters. Blue shows a negative correlation and red shows a positive correlation. * indicates 0.01 < p ≤ 0.05, ** indicates 0.001 < p ≤ 0.01, *** indicates p ≤ 0.001.
